# An Entropy-Based Architecture for Detection of Sepsis in Newborn Cry Diagnostic Systems

**DOI:** 10.3390/e24091194

**Published:** 2022-08-26

**Authors:** Zahra Khalilzad, Yasmina Kheddache, Chakib Tadj

**Affiliations:** Department of Electrical Engineering, École de Technologie Supérieur, Université du Québec, Montréal, QC H3C 1K3, Canada

**Keywords:** newborn cry diagnostic system, Spectral Entropy, sepsis, fuzzy entropy, Bayesian Hyperparameter Optimization

## Abstract

The acoustic characteristics of cries are an exhibition of an infant’s health condition and these characteristics have been acknowledged as indicators for various pathologies. This study focused on the detection of infants suffering from sepsis by developing a simplified design using acoustic features and conventional classifiers. The features for the proposed framework were Mel-frequency Cepstral Coefficients (MFCC), Spectral Entropy Cepstral Coefficients (SENCC) and Spectral Centroid Cepstral Coefficients (SCCC), which were classified through K-nearest Neighborhood (KNN) and Support Vector Machine (SVM) classification methods. The performance of the different combinations of the feature sets was also evaluated based on several measures such as accuracy, F1-score and Matthews Correlation Coefficient (MCC). Bayesian Hyperparameter Optimization (BHPO) was employed to tailor the classifiers uniquely to fit each experiment. The proposed methodology was tested on two datasets of expiratory cries (EXP) and voiced inspiratory cries (INSV). The highest accuracy and F-score were 89.99% and 89.70%, respectively. This framework also implemented a novel feature selection method based on Fuzzy Entropy (FE) as a final experiment. By employing FE, the number of features was reduced by more than 40%, whereas the evaluation measures were not hindered for the EXP dataset and were even enhanced for the INSV dataset. Therefore, it was deduced through these experiments that an entropy-based framework is successful for identifying sepsis in neonates and has the advantage of achieving high performance with conventional machine learning (ML) approaches, which makes it a reliable means for the early diagnosis of sepsis in deprived areas of the world.

## 1. Introduction

Studies conducted by the United Nations Children’s Fund (UNICEF) report that 7000 newborns die every day from mostly treatable causes, which amounts to 2.6 million neonates per year. Although neonates constitute the most vulnerable group, they are also the most difficult to interact with; in-depth examinations and medications are intricate and seldom prescribed. The main challenge in working with neonates is that their only means of communication is crying. According to UNICEF reports, newborn mortality is mainly attributable to infectious pathologies such as sepsis and meningitis. These two pathologic conditions together comprise a 15% share of all neonate death causes, especially in middle and lower-income countries [1].

Crying is the result of cooperation between numerous organs in the body, such as the respiratory system, central and peripheral nervous system, and a variety of muscles and limbs. If any organs fail to function properly, a cry different from a healthy one is expected [2]. As early as the 20th century, it was observed that the cry of neonates diagnosed with certain pathologies was different from healthy neonates [3]. This led to further investigation of cries and the use of sound spectrographic analysis. The results claimed that the cry signal conveys a significant amount of information about a newborn’s health. The researchers developed a more accurate system since the spectrographs could not capture all the abnormalities and disorders in a cry signal; therefore, the automatic newborn cry diagnostic systems (NCDSs) were designed and proposed [4,5,6,7,8,9].

**Figure 1:**
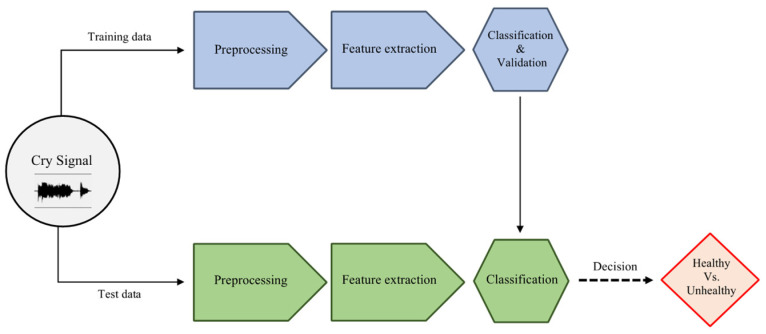
The block diagram of the NCDS.

NCDS architectures are designed to serve different purposes. These purposes include detecting the reason for crying in healthy infants [10,11], such as pain, hunger, etc., segmenting the crying episodes into expiration and inspiration [12], detection of the cry from the surrounding environment [13] and diagnosis of pathologies [14,15,16]. The design proposed in this study focuses on the last category of NCDSs where the goal is to discriminate between healthy and septic infants [17]. Similar to other audio analysis systems, the NCDS consists of three main stages: pre-processing, feature extraction and classification, as seen in Figure 1.

Mel-frequency Cepstral Coefficients (MFCC) are one of the most common features in the analysis of audio signals. They have been employed in the detection of many health conditions, such as cleft palate [18], asphyxia [19,20], respiratory distress syndrome [4] and hearing impairment [21], and have demonstrated efficient performance. Other feature sets, including fundamental and resonant frequencies [22], Linear Prediction Coding (LPC) [23] and prosodic features [24], have been explored in the feature extraction step of other NCDS designs. Various entropy feature sets were utilized in order to identify deaf neonates from the healthy group [21], for detection of asphyxia in newborns [25] and for automated detection of the cry [26]. It has been reported that approximate entropy has different levels across healthy and pathologic newborns [27]. We extracted Spectral Entropy Cepstral Coefficients (SENCC) and Spectral Centroid Cepstral Coefficients (SCCC) and combined them. The combination of these features provides more analysis for the study of septic cry signals. Finally, the feature sets are fed to a classifier and the predicted class labels are the output of the NCDS.

Spectral Centroid (SC) has been studied in order to find the reason for crying [28,29] and to detect infants with developmental disorders [30]. This feature has shown promising results in musical applications for studying timbre [31] and medical studies such as detecting Alzheimer’s disease based on Electroencephalogram (EEG) signals [32]. To the best of our knowledge, cepstral analysis of this feature set has not been explored in NCDS designs so far. For a long time, crying has been treated similarly to the speech signal, and the features that showed potential in speech recognition tasks have been employed in cry research. This study aims to introduce the features that have been prevalent in the study of music to cry-based applications since the cry signal has harmonic components and rhythm [22,24]. In the next step of NCDSs, many different classification approaches have been explored. Support Vector Machine (SVM) [33,34], Probabilistic Neural Network (PNN) [24], Forest [35], Decision Trees [29], K-nearest Neighborhood (KNN) [36], and discriminant analysis are some of the algorithms implemented in this field [37].

Hyperparameter Optimization (HPO) was introduced in the 1990s [38,39] when several studies reported that adjusting various hyperparameters led to better results across different datasets [40]. HPO is employed to enhance the performance of the default settings provided by conventional machine learning (ML) architectures [41,42]. Moreover, Fuzzy Entropy (FE) has been studied previously for many applications in the biomedical field, such as medical database classification [43], and also tested on the Parkinson’s database for feature selection purposes, which was able to achieve an accuracy of 98.28% [44]. 

The contribution in this study has several aspects: first, the identification of septic newborns using their cry signals is of great significance, which has considerable potential and has been rarely looked at so far. To the best of our knowledge, even though sepsis is taking the lives of many newborns every day, there is only one other very recent study dedicated to this pathology. The second contribution is our approach in the design of an NCDS with different feature sets, their combination, and unique HPO for each feature set and classifiers, in order to identify septic newborns. Lastly, we employed a feature selection method based on Fuzzy Entropy (FE Selection) in order to select the features with the highest information content and to reduce the feature space dimensionality [45,46]; to the best of the authors’ knowledge, this method has not been explored in research associated with NCDS so far. There are many other entropy-based features and methods present in the literature. FE selection was chosen for this study due to its simplicity and the fact that it does not burden the system with complex computational costs [47]. Moreover, Lee et al. [45] stated that their FE-based feature selection method enhanced the classification rate by discarding the features that were detrimental and affected by noise. The term *sepsis* refers to an infection that enters the bloodstream. Medical studies suggest that major infections, including sepsis, are associated with tenacious crying, and therefore, for a neonate with persistent crying, the predominant manifestation of sepsis should be seriously considered [48]. Expedient diagnosis is of utmost importance for this pathology and medical staff should be alert to the risk factors of sepsis in neonates [49]. It should be mentioned that there are other effective approaches to the study of sepsis in newborns, which range from studying heart rate monitoring to biosensing and electrochemical detection [50,51]. However, we proposed this study as an early and simple alert for diagnosing sepsis without the need for any clinical equipment, or even contact with the newborn, which would be complementary in adding information regarding sepsis. The areas that suffer the most from septic mortality have a lack of pediatricians and are categorized among low-income countries. Thus, a method that is simple and has efficient performance is preferred to one benefiting from complicated architecture and high computational requirements.

This article aims to provide an automated approach for identifying septic neonates through the development of a Newborn Cry Diagnostic System (NCDS). Furthermore, our goal is to assess the performance of the existing methods in the fields of ML and speech analysis in order to provide a simple tool for early diagnosis of sepsis in infants. It is noteworthy that there are a very limited number of studies dedicated to the automatic identification of septic newborns so far, and we will address them in the following sections. Therefore, there is a lacuna in the studies regarding the automatic analysis of sepsis in neonates. The methodology section explains the data acquisition process, participants and NCDS stages with a detailed description of the features and classifiers. Next, we expound the NCDS evaluation methods and the results in terms of the evaluation metrics are presented. We will then discuss the achieved results and compare them to the work of other researchers. The final section is dedicated to the conclusion.

## 2. Methodology

### 2.1. Cry Dataset and Recording Procedure

The database used for this study was created in collaboration and cooperation with Al-Raee and Al-Sahel hospitals in Lebanon and Saint Justine Hospital in Montreal, Canada. Most of the infants chosen for this study were neonates by the definition of UNICEF, which means they were less than four weeks old. The large number of cases and the diversity of race and pathologies make this database exceptional from all the other databases. The signals were recorded in the hospital environment; they were recorded in different conditions and times, such as after birth, when infants were placed in intensive care units, in the maternity room (either public or private), etc.

The crying reasons were not the same for all the infants; for example, cries may be due to wet diapers, hunger, fear, etc. These reasons were determined according to the conditions causing the cry with the help of medical staff and the infant’s guardians. They were also based on the various tests performed after birth [52]. The dataset acquisition and the selection of the neonates that participated in this study were not limited to a specific cry stimulus, making our study a comprehensive one.

The recorder utilized for this database was an Olympus hand-held digital two-channel device. It had a sampling frequency of 44.1 kHz and 16 bit resolution. The recorder was placed 10 to 30 cm from the newborn’s mouth. There was no well-defined procedure during the acquisition of the cry sounds. Therefore, during the data collection process, unwanted information and noises, such as staff chatter, medical instrument beeps, the cry of the other newborns, and other environmental noises and sounds, were also recorded. Hence, we consider our database a real corpus recorded in an actual clinical environment. Table 1 is a description of the cry database used in this study.

The pathology group selected for this study was sepsis. Our database includes 108 full-term healthy neonates and 17 neonates that were marked as having sepsis by the medical staff through in-depth examinations. There are 53 cry signals recorded from the septic neonates in total, which means each newborn has more than one recording in the database. In order to obtain a balanced study, the same number of samples were chosen from the full-term healthy neonates’ group. The healthy samples were selected completely randomly and without any pre-specified conditions in order to maintain the proposed NCDS free of any bias towards race, reason for crying and origin. In order to have a balanced study, we randomly selected an equal number of samples from both groups. As shown in Table 2, the control group consisted of randomly chosen samples from the whole healthy dataset of 108 healthy newborns to match the number of samples from the septic group. We wanted our NCDS to include newborns from all races, genders and any cry stimuli. The only remaining difference in the two datasets is the number of males and females. However, it has been shown that the length of vocal cords is the factor that determines the fundamental frequency of newborn cries as well as other characteristics, and this is similar across male and female neonates and does not have any meaningful impact on the cry [53]. The average lengths of expiratory and inspiratory cries were 0.72 and 0.21 s, respectively. We set a condition to only select the samples with a length of more than two consecutive windows (17 ms = two 10 ms windows with 30% overlap) in order to achieve a reliable analysis of the dataset.

### 2.2. Dataset Preprocessing

Neonates have no significant control over their cries and therefore can only have a few of the respiratory maneuvers present in adults. Lester et al. [54] reported that the cry pattern of newborns often shows an expiration phase that is five times longer than the inspiration, which was confirmed by the durations of signals for the expiration and voiced inspiration in our dataset.

The process of segmenting and labeling the cry signals was manual and rather perceptive, and consequently a time-consuming one as well. The usual method was to detect the start and end of a cry unit by visual and auditory investigation of the spectrogram of the cry signal [12].

**Figure 2:**
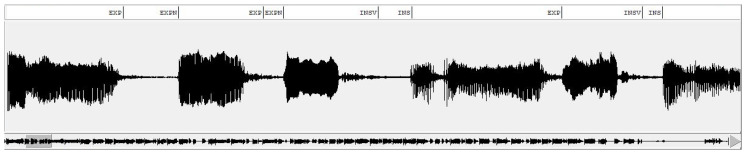
Labels annotated using WaveSurfer software for a cry signal.

Our team of researchers annotated the labels corresponding to various segments of cry signals for this study using WaveSurfer software, as in Figure 2. The recordings of our corpus have been manually annotated to mark the start and endpoints of each vocalization. A newborn cry can comprise typical cry sounds, glottal sounds, hiccups, short pause segments between cries and faint cries [5]. The inspiration is believed to contain information pointing to pain and distress cries [55].

The power needed for driving the expiratory phase of a cry is stored during inspiration. Usually, cries occur during this respiratory phase, so this segment contains the main information [5]. Additionally, voiced inspiration has proven to be significant in the study of pathologic neonates [52]. Therefore, INSV and EXP units are used separately for this study in order to discriminate between healthy and pathologic cries.

### 2.3. Feature Extraction

In the process of generating a cry sound, the impulses produced by the glottis pass through the vocal tract, which acts as a filter. In other words, the vocal tract filters the glottal impulses so as to produce the desired sounds [56]. The Cepstrum is a homomorphic transformation that allows for the discrimination of the source and filter [57]; therefore, cepstral analysis was employed here. Furthermore, the cry signal is non-stationary and dynamic. Hence, an entropy-based feature vector that can capture the presence of complexity in the cry signal is indispensable in the study of newborn pathology diagnosis [58]. Our dataset was recorded in real-world conditions; therefore, the presence of noise was inevitable. In other biological signals, the noise is treated differently based on the purpose and applications [59]. In this regard, as suggested by the previous researchers in our lab [33], we addressed this issue by studying both INSV and EXP datasets in order to be able to have a more reliable representation of the results. Alaie et al. [33] mentioned that EXP cries are more reliable in terms of estimating the true value. Furthermore, the acquisition of the cry signals was done in the same conditions for both healthy and septic newborns, and all the steps for the analysis of both groups were similar. The biological signals are associated with nonstationarities. Maganin et al. [60] reported that these nonstationarities may have detrimental effects on the results. In order to overcome the difficulties in processing and the classification of the nonstationary cry signal, it is standard practice to employ filter banks and a sliding window of short length (10 ms) [61]. The windowing of the nonstationary signal has been introduced as a solution for achieving a locally stationary signal [62]. In this study, the Hamming window and Mel-filter banks were utilized before extracting the features. Each of the introduced feature sets was tested both individually and combined with other features. In the next step, these feature sets were fed to the KNN and SVM classifiers, and the hyperparameters for each of them was optimized using the BHPO method.

#### 2.3.1. Mel-Frequency Cepstral Coefficients (MFCC)

Prior to the extraction of MFCC features, the cry signal needs to be pre-emphasized, which means that the signal is filtered by H(z) = 1 − a$z^{-1}$ as the transfer function of the signal. This filtering allocates higher gains to higher frequencies. In this study, the value of a was selected equal to 0.97 based on previous researchers’ work [33]. Extracting MFCCs consists of four main steps, which are described here [26]:

1. Applying a windowing criterion to the signal: The window was applied to enhance the harmonics, smooth the edges and decrease the edge effect of applying a Discrete Fourier Transform (DFT) to the signal. Here, the Hamming window with a frame size of 10 ms and 30 percent overlap between consecutive frames was selected.
2. Implementing the DFT: In order to obtain the magnitude spectrum of each window, the DFT is applied to the cry signal. In this study, overlapping triangular filters were employed; the number of filters used varied in general between 13 and 24. The MFCC features were computed from 13 filter banks.
3. Computing the logarithm of magnitude and scaling the frequencies on a Mel scale: The magnitude spectrum was multiplied by every triangular Mel weighting filter to calculate the Mel spectrum. The Mel spectrum should be represented on a log scale to be prepared for the next step. Equation (1) gives the Mel scale of frequency *f*.

*M*(*f*) = 1125 *ln*(1 + *f*/700) (1)

4. Taking the inverse Discrete Cosine Transform (iDCT) of the signal: As mentioned before, the energy levels of adjacent bands tend to be correlated due to the smooth form of the vocal tract. Therefore, the transformed Mel-frequency coefficients must undergo an iDCT that results in separable cepstral coefficients. The first few MFCC coefficients might be sufficient for a robust representation of the system [63]. Therefore, the first 13 coefficients were extracted in this study.

MFCCs often only contain the information from one window; hence, these cepstral coefficients are considered static features. In order to gain information on the temporal dynamics, cepstral coefficients’ first and second derivatives should be calculated, which are known as delta and delta-delta coefficients, Equation (2).

(2) $$\Delta_{n}=\frac{\sum_{\theta =1}^{\Theta }\theta \left(c_{n+\theta }-c_{n-\theta }\right)}{2\sum_{\theta =1}^{\Theta }\theta^{2}}$$

where $\Delta_{n}\;$ is a delta coefficient from discrete-time *n* computed in interval of the static coefficients $c_{n-\Theta \;}$ to $c_{n+\Theta \;}$; the value of Θ  is usually set to 2 [61]. The delta-delta coefficients are calculated with delta coefficients in a similar manner. The dynamic features help us capture the spectral changes in the cry signal. Finally, the dynamic MFCC features are added to the feature vector, and together they form the MFCC feature set with a total of 39 features.

#### 2.3.2. Spectral Entropy Cepstral Coefficients (SENCC)

**Figure 3:**
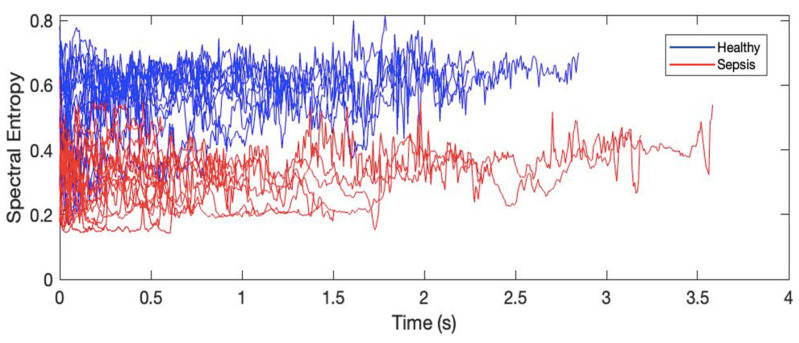
Spectral entropy for 20 EXP utterances from one healthy neonate and 20 EXP utterances from one septic neonate.

Spectral Entropy (SEN) evaluates the signal’s energy distribution uniformity. This measure is an indicator of the complexity of the signal. It can also be employed to capture the peakiness in a signal. Figure 3 illustrates the SEN of multiple episodes of expiration cry for a healthy infant as opposed to an infant diagnosed with sepsis. The entropy levels for a septic cry are lower, which was also deduced in previous works [64].

In order to compute the SEN, the spectrum is written in terms of a Probability Mass Function (PMF)-like function, Equation (3).

(3) $$x_{i}=\frac{X_{i}}{\sum_{i=1}^{N}X_{i}}\;\;\;\mathrm{for}\;i=1\;\mathrm{to}\;N$$

Here, (the uppercase) $X_{i}$, appearing in the nominator and denominator, is the energy of *i*th frequency component of the spectrum. The PMF of the spectrum is represented by (the lowercase) ${x=(x}_{1}{,\ldots ,x}_{N}$), and the number of points in the spectrum is specified by *N*. The entropy of each frame was computed from Equation (4) [65].

(4) $$H=-\sum_{i=1}^{N}x_{i}\;{\cdot \;\log}_{2}x_{i}$$

In order to detect the position of peakiness or flatness present in the spectrum, a process similar to the extraction of the MFCCs was employed. The fast Fourier Transform (FFT) of each frame was calculated. Following the calculation of the FFT, the achieved spectrum was mapped to the Mel-scale in order to mimic the signal based on the human sound perception model. Then, the SEN was computed from the Mel-spectrum. Finally, DCT was applied to decorrelate between the coefficients and further improve the results, and 13 SENCC coefficients were obtained.

#### 2.3.3. Spectral Centroid Cepstral Coefficients (SCCC)

**Figure 4:**
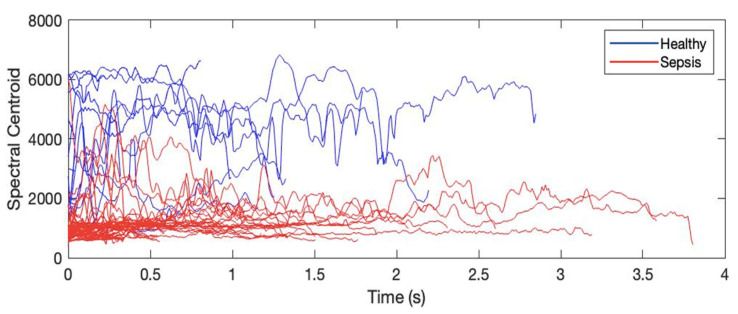
Spectral centroid for 15 EXP utterances from one healthy neonate and 15 EXP utterances from one septic neonate.

SC is a measure of the shape of the spectrum of the signal and the position of the mass of the spectrum. The mean value of SC was shown to be a discriminative feature [66] that indicates where the major energy of the signal is concentrated. SC is expected to be higher for the “brighter sounds” and has been widely employed in the study of timbre for music applications [58]. It is also a discriminative feature in the measurement of tone in audio signals [67]. Figure 4 presents how the cries of the neonates suffering from sepsis are associated with lower tone, as is listed as one of the red-flag listings associated with neonatal sepsis [68].

SC denotes the center of the signal’s gravity and is computed by taking the weighted mean of the frequency bins. The SC value, C_i_ of the *i*-th window, is computed using Equation (5).

(5) $$C_{i}=\frac{\sum_{k=1}^{W_{f_{L}}}{\;\mathrm{kX}}_{i}\left(k\right)}{\sum_{k=1}^{W_{f_{L}}}{\;X}_{i}\left(k\right)}$$

where $x_{i}\left(n\right)$ are the *i*-th window samples, and $X_{i}\left(k\right)$ are the DFT coefficients. The SC cepstral coefficients’ extraction procedure is similar to what was described for MFCC and SENCC, except that for the SCCC feature vector, the first five coefficients were extracted.

### 2.4. Feature Reduction

The first and most crucial aspect of post-processing is to reduce the dimensionality of the feature vectors to decrease the storage and computational costs. Feature reduction includes all the techniques that aim to make a compact feature set out of the original sets while trying to keep as much information as possible. Camargo et al. [69] suggested a simple and rapid method that reduces data through statistical operations such as minimum, maximum, average and standard deviation. Messaoud et al. [7] also proposed an arithmetic method by averaging MFCCs over a time axis. Matikolaie et al. [4] further investigated the use of statistical methods in the compression of the MFCC feature set and reported that this method was effective in terms of computational costs and classification accuracy. In order to reduce the dimensionality of the MFCC feature set, the statistical approach was employed, and the mean value of each MFCC coefficient over the time axis of each signal was calculated.

### 2.5. Fuzzy Entropy Based Feature Selection

As explained in the previous sections, entropy is associated with the uncertainty of a given variable. Here, we aim to focus on the concept of fuzzy entropy, which calculates entropy through a fuzzy c-means clustering algorithm. This method is called Fuzzy Entropy Selection of the features (FE Selection). In general, fuzziness refers to a possibilistic point of view, while the aforementioned entropy measure focuses on randomness and has a probabilistic perspective. This method was chosen because it is very fast and imposes a negligible computational cost on the system [47].

Trivedi et al. [70] introduced a Fuzzy c-Partition model that computed the membership of each feature dimension and its corresponding FE. Suppose a finite set where Y = {$y_{1}$, $y_{2}$, …, $y_{n}$}, a set of real c×n matrices denoted by $V_{cn}$, and *c* is an integer so that 2≤c<n. The fuzzy c-partition space, $M_{fc}$, for Y is given by Equation (6).

(6) $$M_{fc}=\left\{U\in V_{cn}|\;u_{ik}\in \left[0,\;1\right],\;\forall i,k;\sum_{i=1}^{c}u_{ik}=1,\;\forall k;\;0<\sum_{i=1}^{c}u_{ik}<n,\forall i\right\}$$

This means that membership values of $y_{j}\;$ in the *c* subsets could be obtained from the *j*th column of matrix *U*, which is from c×n dimensions. The grade of membership of $y_{k}$ in the *i*th fuzzy subset of Y is represented by $u_{ik}=u_{i}\left(y_{k}\right)$. Therefore, the membership of each pattern $y_{k}$ in all subsets is calculated and then normalized. Instead of applying this algorithm to each pattern, it is applied to each feature similar to previous studies [47]. The *FE* is calculated based on the matching degree, $D_{c}$, described by Equation (7), where $u_{c}$ is the membership of the feature $y_{d}\;$ in each of our two classes, denoted by *c* for each class and *C* for the set of the two classes [45].

(7) $$D_{c}=\frac{\sum_{y_{d}\in c}u_{c}\;\left(y_{d}\right)}{\sum_{y\in C}u_{c}\;\left(y_{d}\right)}$$

The *FE* of the elements of each of these classes is achieved through Equation (8).

(8) $$FE_{c}=-D_{c}\log D_{c}$$

Finally, the overall *FE* is given by Equation (9):

(9) $$FE=\sum_{c=1}^{C}FE_{c}=FE_{Healthy}+FE_{Septic}$$

The main interpretation of the *FE* is very similar to the SEN which was described before; higher entropy translates to lower information content. We based our feature selection on the fact that smaller *FE* values contribute more to the recognition of septic infants. Thus, we first calculated the average *FE* value across the features and set this value as a threshold for our feature selection. In the next step, we imposed a condition where only the features with *FE* values lower than the overall average *FE* should be selected and formed a new feature set to be fed into the classifier. This condition secures the selection of features with minimum overlap and also will likely result in a lower misclassification possibility, which will be evaluated by the Matthews Correlation Coefficient (MCC) measure.

### 2.6. Classification

The performance of the feature sets was tested by the two classification methods of KNN and SVM in order to discriminate between the healthy and septic neonates. Each EXP or INSV cry episode was treated as a sample and the classifier assigned a label of healthy or septic to it. Both classification methods benefit from five-fold cross-validation in order to avoid over-fitting and ensure credibility. The models were tuned with the BHPO method in order to enhance the performance of each model.

#### 2.6.1. K-Nearest Neighborhood (KNN)

This method is an efficient yet simple method of classifying data. As the name of this method suggests, the features with similar values belong to the same class. The KNN classifiers often use Euclidean distance for the measurement of the distance between data points. This classifier has three bases for classification: sets of labeled data, a distance measure and, finally, the number of neighbors, which is denoted by K. In other words, KNN classifies a given sample based on the majority vote of the neighborhood and the distance [71,72]. The number of neighbours was automatically tuned with the BHPO method in the first step, which in all of the given experiments returned K = 1 as the best choice. The other hyperparameter selected for tuning is the type of distance used with each feature set. The distance measures included in this optimization include Minkowski, Chebyshev, Euclidean, standard Euclidean, cosine, Jaccard, Manhattan and Hamming.

#### 2.6.2. Support Vector Machine (SVM)

SVM has a broad application in the classification of audio signals. An SVM differentiates between two cases by implementing a hyperplane. SVM is inspired by the statistical learning theory and the Vapnik–Chervonenkis (VC) dimension. The optimal hyperplane is constructed when the distance between the hyperplane and data is considerable. The linear data can be classified by simply constructing a straight hyperplane, while the nonlinear data should be made linearly separable for the purpose of classification. It means that the data must pass through a transformation into high-dimensional space, which is known as the kernel function [73]. The gaussian kernel is used in this study. The hyperparameters selected for HPO were kernel scale and box constraint. The BHPO was used for the tuning of the mentioned hyperparameters of the SVM model as well.

#### 2.6.3. Bayesian Hyperparameter Optimization (BHPO)

In order to maintain the classification errors at a minimum while achieving high performance in a ML problem, HPO methods are used. A majority of ML designs include hyperparameters. With recent advances in the field of automated ML, various methods such as random search, grid search and Bayesian optimization have been introduced that no longer require human efforts for tuning these hyperparameters. More importantly, the hyperparameters are tailored to meet the requirements of each specific task and the results are reproducible. The basis of HPO is finding the optimal value for the hyperparameters in a finite set of predefined values, in order to minimize or maximize an objective function (e.g., model performance). The common challenge with these grid search and random search methods is the high number (~90 iterations) of function evaluations needed to obtain minimal error, which in turn is not cost-effective and may cause curse of dimensionality [74]. BHPO is also an iterative method in which the acquisition function and the probabilistic surrogate model are the vital elements. The model is constantly updated based on the objective function evaluation, which is expressed as Equation (10) [75]:

(10) $$x^{*}=\underset{x\in X}{argmin}f\left(x\right)$$

The methodology in summary is deduction of the information on the model in each iteration based on new hyperparameters and the resulting model performance. When the number of determined iterations ends, the global optimal hyperparameter configuration is reported. In order to establish the local optimal hyperparameter, the acquisition function employs the predictive information of each possible hyperparameter configuration. BHPO requires far fewer iterations when compared to the other two methods and all the experiments in this study were performed with only 30 iterations.

## 3. Evaluation and Results

The features introduced in this study were extracted and fed to the classifiers with the purpose of distinguishing between healthy and septic neonates. In order to compare their abilities to reach that goal, several experiments were conducted which were comprised of different feature sets, implementing the features individually or combined, and two classification methods with a wide range of parameters. Finally, the models were tuned to obtain the best performance. In this framework, the following feature sets were used:

- MFCC;
- SENCC;
- SCCC;
- MFCC + SENCC;
- SENCC + SCCC;
- MFCC + SCCC;
- MFCC + SENCC + SCCC.

Five-fold cross-validation was carried out after feeding each feature set to the classifier. This means that one fold of data was treated as the test data in each iteration of the training process, and the other four were the training folds. This process was repeated until all the folds had been used as the test fold. This process was repeated for both EXP and INSV datasets.

### 3.1. Evaluation Criteria

**Figure 5:**
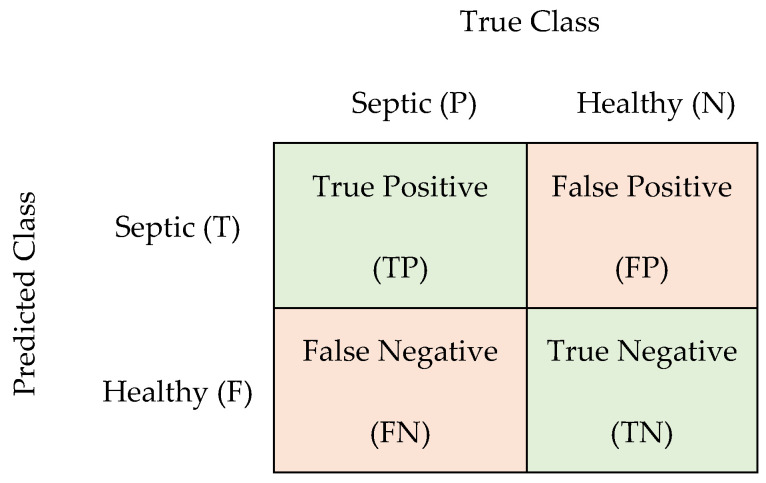
The confusion matrix for a binary classification.

There are different approaches to evaluating a system’s performance. One of the main measures for that purpose is accuracy. Accuracy is the ratio of correct decisions to the total number of cases, Equation (11).

(11) $$\mathrm{Acc}=\frac{\mathrm{TP}+\mathrm{TN}}{\mathrm{TP}+\mathrm{TN}+\mathrm{FN}+\mathrm{FP}}$$

where N stands for negative and P stands for positive, and T and F stand for true and false. However, when the task is diagnosing a pathology, it is of utmost importance that the system does not miss a pathologic case. A confusion matrix is defined for the binary classification task where the problem is the discrimination between healthy and pathologic cries, as shown in Figure 5. In this study, the positive label stands for septic infants and the negative label stands for healthy (not septic).

The True Positive Rate (TPR) is referred to as sensitivity, hit rate or recall. In the concept of this study, recall is also an important measure as it demonstrates how many true septic cases have been captured by the NCDS. Hence, recall owes its importance to the fact that a false healthy detection is not desirable, Equation (12) [76].

(12) $$\mathrm{TPR}=\frac{\mathrm{TP}}{\mathrm{TP}+\mathrm{FN}}$$

The Positive Predictive Value (PPV) is another measure and is also referred to as precision. In this framework, precision is the probability that a septic case is predicted as septic, Equation (13).

(13) $$\mathrm{PPV}=\frac{\mathrm{TP}}{\mathrm{TP}+\mathrm{FP}}$$

The next evaluation measure is called the F1-score, which shows the balance between precision and recall and is a good measure of the system’s performance. Mathematically, the F1-score is the harmonic mean of precision and recall, Equation (14).

(14) $$F1=\frac{\mathrm{TP}}{\mathrm{TP}+0.5\;\left(\;\mathrm{FP}+\mathrm{FN}\right)}=2\cdot \frac{\mathrm{precision}\;\cdot \;\mathrm{recall}}{\mathrm{precision}+\mathrm{recall}}$$

Finally, the MCC considers all the information in a contingency matrix. The value of this measure belongs to the [−1, +1] interval where 0 denotes a random distribution, −1 shows complete misclassification and +1 corresponds to perfect classification [77].

The MCC is computed using Equation (15):

(15) $$\mathrm{MCC}=\frac{\mathrm{TP}\;\times \mathrm{TN}-\mathrm{FP}\times \mathrm{FN}}{\sqrt{\left(\mathrm{TP}+\mathrm{FN}\right)\left(\mathrm{TN}+\mathrm{FP}\right)\left(\mathrm{TP}+\mathrm{FP}\right)\left(\mathrm{TN}+\mathrm{FN}\right)}}$$

The MCC measure is highly informative for binary classification tasks in general [78]. Since we have a healthy versus septic classification problem in this study, implementing the MCC is considered beneficial and proper.

### 3.2. Results

The results of different experiments conducted in this study are given in Table 3, Table 4, Table 5, Table 6, Table 7, Table 8, Table 9 and Table 10. As previously mentioned, we analyzed the performance of feature sets for two separate datasets of EXP and INSV. Moreover, KNN and SVM were employed as the classifiers in this study. The feature sets were used both individually and jointly. They were concatenated so that we could compare the performance of larger feature sets as opposed to the individual feature sets. It is noteworthy that our findings regarding the behavior of feature sets were consistent with medical findings and other researchers’ work, as discussed in Section 2.3.2 and Section 2.3.3. Regarding the evaluation criteria discussed in the previous section, the higher the value of each measure, the better the performance of our NCDS. The results presented in this section are all in the form of average and standard deviation of five-fold cross validation values. For all the measures, the values represent percentages except for the MCC measure, which is unitless and belongs to the [−1, 1] range.

Table 3 presents the results for the evaluation of the MFCC feature set for EXP and INSV datasets. Furthermore, the MFCC feature set was evaluated with the use of the HPO method. We used BHPO for both classifiers, as mentioned in the previous sections. Finally, the performance of this feature set was tested with the KNN and SVM classifiers. The HPO led to consistent enhancement of accuracy and F-score measures across both datasets for the MFCC feature set. The SVM classifier had better performance in the evaluation of the MFCC feature set in both datasets in terms of all the evaluation measures except for recall, where the KNN classifier showed better performance. The best results achieved by this feature set are highlighted.

Overall, the highest achieved F-score and accuracy for the EXP dataset were 88.07% and 87.66%, respectively. In this regard, the performance of the NCDS with the INSV dataset was superior to the EXP dataset; the highest overall results obtained for this dataset in terms of F-score and accuracy were 89.06% and 89.13%, respectively.

As can be seen in Table 4 and Table 5, the performance of our NCDS with the SENCC and the SCCC feature sets were similar; both feature sets achieved 72.02% accuracy measures (with different standard deviations). Furthermore, the SENCC and the SCCC feature sets obtained 61.33% and 61.71%, respectively, for F-score with the KNN classifier for the EXP dataset. Also, both datasets and feature sets obtained 100% precision and specificity with the SVM classifier. In the evaluation of the INSV dataset, KNN had better performance in terms of accuracy and F-score. The best F-score for the SENCC dataset was achieved with the KNN classifier for the INSV dataset, which was equal to 62.15%. Regarding the SCCC feature set, the highest F-score was 61.71% for the EXP dataset using the KNN classification method.

In the next step, the framework of feature combination was investigated. We examined all possible combinations of these feature sets that were made possible through their concatenation. The results of these combinations are presented in Table 6, Table 7, Table 8 and Table 9. It can be observed that using the SVM classification method, the combination of SENCC and SCCC was dominated by the SENCC feature set for the EXP dataset and by SCCC for the INSV method since, despite the difference in their kernel scales, there was not a change in the evaluation measures. The overall best accuracy and F-score for the combination of SCCC and SENCC belonged to the KNN classification of the EXP dataset with 72.52% and 63.23%, respectively.

The addition of the SCCC feature set to the MFCC feature set with the SVM classifier achieved the results of 88.41% and 88.25% for accuracy and F-score measures with the INSV dataset, as seen in Table 7. Furthermore, using the KNN classifier with the EXP dataset resulted in better performance in terms of accuracy and F-score, with 82.44% and 83.39%, respectively.

As can be interpreted from Table 8, the best performance in terms of accuracy and F-score measures for the EXP dataset across all the experiments was achieved by the combination of the MFCC and SENCC feature sets. The highest accuracy and F-score among all the experiments on the EXP were 89.99% and 89.70%, respectively. Regarding the EXP dataset, the accuracy and F-score measures were enhanced by 1.92% and 2.04%, respectively, compared to the MFCC feature set, which had the highest accuracy and F-score among the individual datasets.

Finally, the combination of all the individual feature sets with the SVM classification resulted in the highest accuracy and F-score across all the experiments for the INSV dataset, with 89.42% for both measures, as seen in Table 9. The combination of all individual feature sets enhanced these two measures by 0.36% and 3.31%, respectively, compared to the MFCC feature set, which achieved the best results among the individual feature sets.

As our final experiment, we computed the FE measure for the best two experiments discussed above and selected the most compatible features in each presented feature set. These two experiments included the combination of the MFCC and SENCC features for the EXP dataset and the combination of all features for the INSV dataset, both classified using the SVM method. Table 10 represents the results of applying the FE selection method to these two experiments.

**Figure 6:**
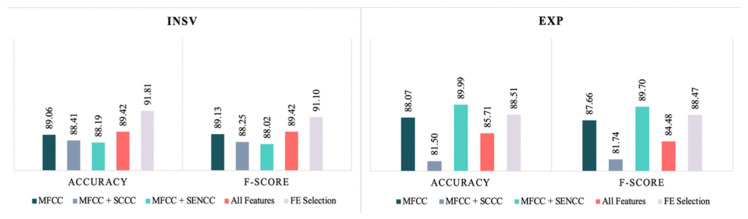
Best F-score and accuracy measures for the SVM classifier in each feature set.

According to the evaluation measures studied here, the FE selection method was highly successful. Implementing fewer features resulted in a negligible decrease in the evaluation measures for the EXP dataset. As for the INSV dataset, the FE selection led to enhancement of all the evaluation measures, which marked the highest accuracy and F-score measures across all the experiments with 91.81% and 91.10%, respectively. Figure 6 summarizes the results of the experiments in terms of F-score and accuracy measures for the SVM classifier that yielded the best results for a clearer comparison. 

## 4. Discussion

This study further explored sepsis in newborns by the means of studying their cry signal through developing an NCDS design. Even though sepsis is associated with high mortality rates in newborns, only one recent work in our lab has studied the cries of septic infants in parallel to the study presented here. The previous study in our lab did not discuss the performance of the system in terms of the accuracy measure [37]. In this study, accuracy as well as several other evaluation measures were included to help better study the performance of NCDSs for diagnosing septic newborns. Our goal was to build upon the previous work and also design a simple model that could achieve improved or comparable performance. Moreover, it is worth highlighting this research’s novelty in terms of analyzing the infant cry from the perspective of musical machine-learning applications. Most of the works addressing infant cries have treated the cry signal as a pre-speech audio. We believed that the harmonic nature of the infant cry, as well as the natural differences in the voice generation organs of infants and adults, had the potential to be analyzed with the features and methods that have shown promising results in the field of musical signal processing. There is meager information on the behaviour of pathologic cries based on analysis of the SC, and this work is the only study that combines SC with cepstral analysis in the study of pathologic newborn cries.

Nowadays, many audio recognition system designs benefit from state-of-the-art deep learning and ML methods. However, the main challenge in studying pathology-related applications is the acquisition of relevant data. The occurrence of a specific pathology in any given time interval in newborns is not predictable and meeting the ethical and technical requirements to include cry samples in a database calls for extreme measures. Therefore, this study explored different approaches to make the best use of the available data. The limitations of the data impose many challenges in NCDS design. Inspired by [37], we also addressed this issue by segmenting each cry signal into multiple expiratory and inspiratory episodes in order to treat each segment as a sample. Despite our efforts to make the analysis in this study unbiased towards race, origin and other factors, it should be noted that the system might still suffer from a low generalization power since it was designed based on a limited number of participants. Therefore, future research should be devoted to further investigate this matter. Moreover, the data dimensionality imposed more challenges in the process of feature extraction. It is common practice in NCDS studies to use statistical measures with extracted features to reduce computational costs [4,7]. The statistical method was chosen to ensure that our results are comparable to the previous studies. Furthermore, extra attention should be paid to the details in the design of conventional models because limited data may lead to overfitting of the classifiers. We addressed this challenge by using BHPO for both the SVM and KNN classification methods. As can be interpreted from Table 6, the accuracy of the NCDS was enhanced up to 89.42% for the INSV dataset. Also, we believed that the characteristics that were reported in the medical studies conducted on septic cries could be better analyzed through cepstral analysis of the SC and the SEN features, which was confirmed by our findings. Through the implementation of these features, the presented work was made capable of obtaining F-scores of 89.70% for the EXP dataset and 89.42% for the INSV dataset, which were both superior to the previous study [37]. Therefore, we were able to show that even a single episode (as opposed to the All Episode voting scheme) analysis of the cry signal could achieve reassuring performance with careful selection of the parameters.

As mentioned, the performance of the system was tested with the two different classification approaches of SVM and KNN, and SVM showed superiority in a majority of experiments. The recall measure was an exception to this conclusion, where KNN showed better performance. The presented study also showed that elevating the number of features in a pattern recognition problem does not always enhance the system’s performance. The predictive performance of the system depends on many different factors.

As was mentioned previously, the high discriminative power of inspiratory cries in the study of pathologic newborns has been neglected in many works. However, the high values of the evaluation measures achieved for this dataset show the potential for further investigation of inspiratory cries, which was consistent with previous studies in our lab.

As discussed in Section 3, the entropy levels differ across healthy and septic infants, which is also reported by other researchers where healthy newborn cries were distinguished from pathologic cries [27]. The same explanation applies to the SC of the infant cries, which marks these feature sets as potential biomarkers for further study of septic newborns. The SENCC measure alone could achieve 72% accuracy with the SVM classifier; it yields the highest performance in this study when combined with the MFCC feature sets.

**Figure 7:**
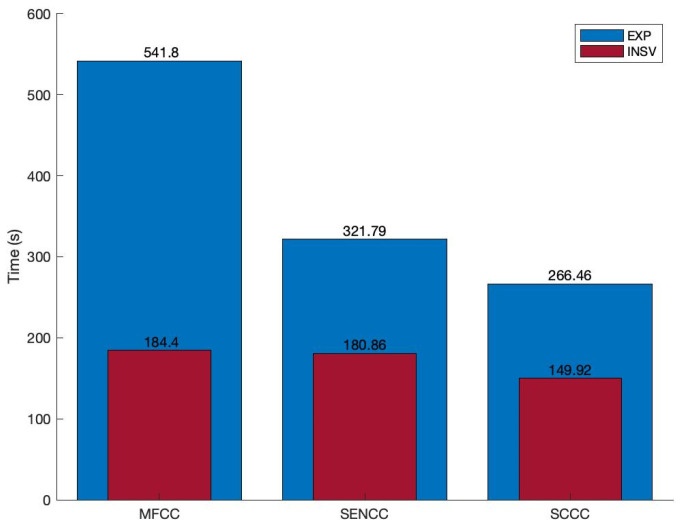
The elapsed time for the extraction of features.

Figure 7 shows the elapsed time for extracting each of our feature sets for EXP and INSV datasets. The elapsed times are rational in terms of the duration of datasets and the number of coefficients in each feature set. Nevertheless, it was validated that extracting the SENCC and SCCC features does not aggravate the system’s complexity in terms of computational costs, and they have similar performance and run-times.

It has been reported that the aggregation of multiple classifiers, with the intention of having the classifiers compensate for the errors of each other, does not yield good results and only burdens the system with more complexity and computational cost [37]. In order to overcome this issue, we utilized BHPO with only 30 iterations, which is a low-cost and fast method. We were able to outperform the mentioned model in terms of F-score by between 3–6% for both datasets.

None of the conducted experiments showed misclassification in terms of the MCC measure since they all had positive values. Moreover, all the combined feature sets for the EXP dataset yielded MCC values higher than 0.50. MCC values consider all elements from a confusion matrix; thus, their high value means prediction had satisfactory performance in terms of TP, TN, FN and FP. The same explanation applies to the INSV dataset, except for the feature set formed by the combination of the SENCC and SCCC features.

**Figure 8:**
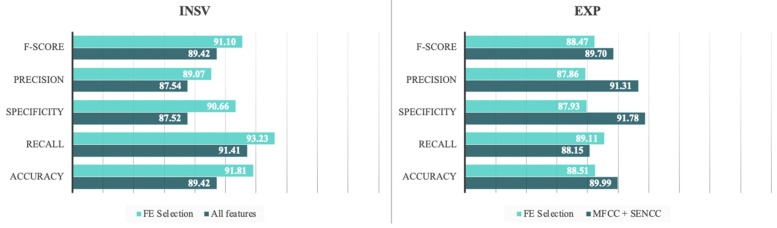
The comparison of results before and after applying the FE Selection method.

As a final contribution, we further explored the use of entropy-based measures in the framework of diagnosing pathologies in infants based on their cry signals. By calculating the FE of the combined feature sets, we were able to remove redundant features, and also identified which features yielded better information in the feature set. After calculating the average FE across all measures, we set a threshold for the selection of the features and removed all the features with a higher FE value than the average. As a result, the system’s accuracy for the EXP dataset was not notably hindered by removing more than 40% of the features, and it was even enhanced in terms of the recall measure. Moreover, all of the evaluation measures were enhanced for the INSV dataset, which shows the reliability of this feature selection method in selecting the most prominent features. Figure 8 shows the difference in the evaluation measures for the best experiments in each dataset, after removing nearly 50% of the features based on their FE.

The results from these experiments also highlighted the fact that incrementing the number of features may not always lead to higher accuracy or enhanced performance of the system. Furthermore, it is noteworthy that understanding the information content of the feature space and selection of the most compatible features accordingly improves the performance of the system, as seen through the INSV dataset experiments where using FE selection enhanced the system’s performance by an average of 2%.

As discussed before, high recall values show the ability of the NCDS in the successful detection of septic cases. The MFCC feature set had the best performance in terms of recall among all the individual feature sets with 92.74% for the INSV dataset. The overall highest recall was obtained by combining all feature sets for the INSV dataset with 94.22%.

The implementation of the FE was a successful experiment in addition to all other presented experiments on the septic newborn cry signals. Our main achievement through the study of FE was to reduce the feature space by more than 40% while keeping the same performance; however, the improvement from the FE alone was limited. This experiment was simply carried out to evaluate if the system could benefit from further simplification and to eliminate the features corrupted by noise. We tried to develop each stage of the proposed NCDS in a way that was not explored well enough or not investigated in the field of NCDS designs. This included the analysis of septic newborn cries in NCDSs for only the second time ever, introducing the use of cepstral coefficients of entropy and centroid to NCDS design, the ways we manipulated these features in order to study the newborn cries, the use of FE for feature selection, and employing BHPO for both the SVM and KNN methods, all of which, to the best of our knowledge, was unprecedented in NCDSs. We acknowledge that the study presented here cannot cover all aspects of the study of septic newborn cries and may be improved upon in many ways. There is an unceasing need for more studies in this field. The authors suggest exploring more classification schemes such as naïve Bayesian, Ensemble classifier, etc., and fusing their outcomes to form a more precise decision. There are more in-depth ideas for investigation that can assess the effect of the inevitable noise in the biological signals, as well as exploring other entropy-based measures, which could not be explored in the scope of this study.

## 5. Conclusions

In the presented study, sepsis was targeted as one of the leading mortality causes of neonates worldwide. The main goal was to develop a simple NCDS which is capable of detecting septic infants without the need for in-depth and invasive clinical tests. The recording of the cries does not need any complicated equipment, it can be done with a commercial handheld recorder, and it does not require any special conditions (our database was recorded in maternity rooms, NICUs, etc.). It does not even necessitate touching the newborn. We believed it was worth exploring how the cries of septic newborns would be different from those of healthy newborns as a complementary method to other means present in the literature. The novelty of our proposed work is in taking common tools in audio, music and speech processing, combining them, and tuning them in such a way that the final design is still simple but is able to achieve high performance in comparison to the other similar methods that are computationally expensive. The proposed NCDS could be employed as an early alarm for medical staff to detect possible pathologic neonates as soon as possible. Within this framework, entropy was utilized in various stages of the architecture, and yet it avoided complicated designs as well as any need for high-end technologies. We studied the infant cries with a musical perspective by employing SEN and SC features and their combination with cepstral analysis. These feature sets were classified using KNN and SVM classifiers that were tuned specifically for each of the feature sets and datasets by the BHPO methods. We also introduced a FE feature selection framework for the first time in the study of pathologic infant cry signals. By using this method, we further simplified our NCDS design and removed nearly half of the redundant, low-impact and noise-affected features. The performance of our design was evaluated using two separate datasets of expiratory cries (EXP) and inspiratory cries (INSV) with various evaluation measures such as accuracy, F-score and MCC. The achieved results showed promising potential in every step of the study. Each stage of the design further improved the system’s performance, at least in terms of one of the evaluation metrics. The best results in terms of accuracy and F-score measures were achieved by combining all the introduced features after FE selection for the INSV dataset with the SVM classifier, and these were 91.10% and 91.81%, respectively. These results also highlight the importance of INSV cries as potential biomarkers, which has been neglected in many infant cry studies. Finally, we concluded that the framework presented here has promising potential in studying and diagnosing sepsis in newborns all around the world as a non-invasive means, especially in areas that are facing challenges with a lack of experts and specialists.

For a list of all acronyms, please see Appendix A.

## Figures and Tables

**Table 1:** Description of the cry database.

	Septic	Healthy
**Gender**	11 Males and 6 Females	55 Females and 53 Males
**Weight**	3.03 ± 0.40 kg	3.50 ± 0.55 kg
**APGAR Score**	8 to 10, measured 2–3 times	9–10, measured 2–3 times
**Babies’ Ages**	1 to 53 days old	
**Prematurity**	Full term	
**Gestational Age**	38 ± 1 week	
**Origin**	Canada, Haiti, Portugal, Syria, Lebanon, Algeria, Palestine, Bangladesh, Turkey	
**Race**	Caucasian, Arabic, Asian, Latino, African, Native Hawaiian, Quebec	
**Reason for Crying**	Birth cry, hunger, dirty diaper, discomfort, needs to sleep, cold, pain	

**Table 2:** Specifications of EXP and INSV datasets for healthy and pathologic cry signals.

	No. of Healthy	No. of Septic	No. of Train Samples	No. of Test Samples	Available Time (s)
**EXP**	1132	1132	1585	679	1773.66
**INSV**	461	461	646	276	442.27

**Table 3:** Evaluation metrics for the MFCC feature set.

MFCC	EXP		INSV	
	SVM	KNN	SVM	KNN
Accuracy (%)	88.07 ± 0.98	81.97 ± 0.70	89.06 ± 1.80	85.36 ± 0.49
Recall (%)	85.71 ± 1.91	91.67 ± 1.83	91.85 ± 2.96	92.74 ± 0.33
Precision (%)	90.38 ± 0.74	72.48 ± 1.93	86.38 ± 1.05	78.30 ± 0.81
Specificity (%)	89.72 ± 0.72	76.56 ± 1.01	86.58 ± 1.16	80.36 ± 0.61
F-score (%)	87.66 ± 1.13	83.42 ± 0.67	89.13 ± 1.90	86.11 ± 0.42
MCC	0.76 ± 0.02	0.65 ± 0.01	0.78 ± 0.04	0.72 ± 0.01
Distance/Kernel Scale	1.7864	Cosine	5.8165	Cosine

**Table 4:** Evaluation metrics for the SENCC feature set.

SENCC	EXP		INSV	
	SVM	KNN	SVM	KNN
Accuracy (%)	71.55 ± 0.70	72.02 ± 0.82	69.20 ± 0.85	65.00 ± 1.71
Recall (%)	42.50 ± 1.42	44.88 ± 1.85	37.04 ± 1.74	58.81 ± 3.13
Precision (%)	100.00 ± 0.00	98.60 ± 0.24	100.00 ± 0.00	70.92 ± 2.01
Specificity (%)	100.00 ± 0.00	96.93 ± 0.43	100.00 ± 0.00	65.95 ± 1.82
F-score (%)	59.64 ± 1.40	61.33 ± 1.68	54.04 ± 1.84	62.15 ± 2.29
MCC	0.52 ± 0.01	0.52 ± 0.01	0.48 ± 0.01	0.30 ± 0.03
Distance/Kernel Scale	0.0116	Cosine	0.1063	Chebyshev

**Table 5:** Evaluation metrics for the SCCC feature set.

SCCC	EXP		INSV	
	SVM	KNN	SVM	KNN
Accuracy (%)	71.46 ± 0.67	72.02 ± 0.77	69.06 ± 0.83	68.12 ± 0.87
Recall (%)	42.32 ± 1.35	45.60 ± 1.73	36.74 ± 1.71	37.33 ± 1.93
Precision (%)	100 ± 0.00	97.90 ± 0.24	100.00 ± 0.00	96.03 ± 0.81
Specificity (%)	100 ± 0.00	95.52 ± 0.39	100.00 ± 0.00	90.04 ± 1.72
F-score (%)	59.46 ± 1.33	61.71 ± 1.55	53.72 ± 1.82	52.75 ± 1.91
MCC	0.52 ± 0.01	0.51 ± 0.01	0.48 ± 0.01	0.41 ± 0.02
Distance/Kernel Scale	0.0089	Jaccard	0.0129	Hamming

**Table 6:** Evaluation metrics for the combination of SCCC and SENCC feature set.

SCCC + SENCC	EXP		INSV	
	SVM	KNN	SVM	KNN
Accuracy (%)	71.55 ± 0.70	72.52 ± 0.89	69.06 ± 0.83	65.72 ± 1.24
Recall (%)	42.50 ± 1.42	47.80 ± 2.13	36.74 ± 1.71	58.52 ± 2.10
Precision (%)	100.00 ± 0.00	96.73 ± 0.38	100.00 ± 0.00	72.62 ± 2.28
Specificity (%)	100.00 ± 0.00	93.50 ± 0.48	100.00 ± 0.00	67.21 ± 1.68
F-score (%)	59.64 ± 1.40	63.23 ± 1.79	53.72 ± 1.82	62.54 ± 1.47
MCC	0.52 ± 0.01	0.51 ± 0.01	0.48 ± 0.01	0.31 ± 0.03
Distance/Kernel Scale	0.0951	Jaccard	0.0764	Cosine

**Table 7:** Evaluation metrics for the combination of SCCC and MFCC feature set.

MFCC + SCCC	EXP		INSV	
	SVM	KNN	SVM	KNN
Accuracy (%)	81.50 ± 1.46	82.44 ± 0.65	88.41 ± 1.77	87.25 ± 0.94
Recall (%)	83.69 ± 2.40	89.05 ± 1.42	89.19 ± 3.25	92.74 ± 1.61
Precision (%)	79.36 ± 1.53	75.98 ± 0.98	87.66 ± 1.47	81.99 ± 2.28
Specificity (%)	79.89 ± 1.31	78.41 ± 0.62	87.38 ± 1.39	83.17 ± 1.62
F-score (%)	81.74 ± 1.57	83.39 ± 0.69	88.25 ± 1.92	87.68 ± 0.84
MCC	0.63 ± 0.03	0.66 ± 0.01	0.77 ± 0.04	0.75 ± 0.02
Distance/Kernel Scale	6.5705	Standard Euclidean	2.5893	Manhattan

**Table 8:** Evaluation metrics for the combination of SENCC and SENCC feature set.

MFCC + SENCC	EXP		INSV	
	SVM	KNN	SVM	KNN
Accuracy (%)	89.99 ± 0.71	86.83 ± 0.44	88.19 ± 1.42	84.57 ± 0.75
Recall (%)	88.15 ± 1.75	91.07 ± 0.87	88.89 ± 3.31	90.07 ± 0.66
Precision (%)	91.78 ± 0.75	82.68 ± 1.56	87.52 ± 1.19	79.29 ± 0.92
Specificity (%)	91.31 ± 0.65	83.76 ± 1.10	87.22 ± 0.94	80.64 ± 0.79
F-score (%)	89.70 ± 0.83	87.26 ± 0.31	88.02 ± 1.61	85.10 ± 0.70
MCC	0.80 ± 0.01	0.74 ± 0.01	0.76 ± 0.03	0.70 ± 0.01
Distance/Kernel Scale	2.1612	Minkowski	4.5656	Correlation

**Table 9:** Evaluation metrics for the combination of all feature sets.

All Features	EXP		INSV	
	SVM	KNN	SVM	KNN
Accuracy (%)	85.71 ± 1.17	82.77 ± 0.29	89.42 ± 1.01	85.87 ± 0.92
Recall (%)	78.75 ± 3.34	85.03 ± 1.54	91.41 ± 1.62	94.22 ± 0.97
Precision (%)	92.54 ± 1.26	80.29 ± 1.51	87.52 ± 1.63	77.87 ± 1.36
Specificity (%)	91.21 ± 1.04	80.93 ± 0.93	87.54 ± 1.42	80.31 ± 1.02
F-score (%)	84.48 ± 1.62	83.05 ± 0.38	89.42 ± 1.00	86.71 ± 0.84
MCC	0.72 ± 0.02	0.66 ± 0.01	0.79 ± 0.02	0.73 ± 0.02
Distance/Kernel Scale	2.3092	Euclidean	3.8005	Cosine

**Table 10:** Evaluation metrics after applying FE Selection to the best feature sets of previous experiments.

FE Selection	EXP: MFCC + SENCC		INSV: All Features Combined	
	All	FE Selection	All	FE Selection
Accuracy (%)	89.99 ± 0.71	88.51 ± 0.77	89.42 ± 1.01	91.81 ± 0.75
Recall (%)	88.15 ± 1.75	89.11 ± 1.32	91.41 ± 1.62	93.23 ± 0.44
Precision (%)	91.78 ± 0.75	87.93 ± 0.84	87.52 ± 1.63	90.66 ± 1.18
Specificity (%)	91.31 ± 0.65	87.86 ± 0.76	87.54 ± 1.42	89.07 ± 1.25
F-score (%)	89.70 ± 0.83	88.47 ± 0.81	89.42 ± 1.00	91.10 ± 0.77
MCC	0.80 ± 0.01	0.77 ± 0.02	0.79 ± 0.02	0.84 ± 0.01
Number of Features	52	27	57	35

## Data Availability

Not applicable.

## Appendix A

**Table A1:** List of acronyms.

Full Name	Acronym
Mel-frequency Cepstral Coefficients	MFCC
Spectral Entropy Cepstral Coefficients	SENCC
Spectral Centroid Cepstral Coefficients	SCCC
Electroencephalogram	EEG
K-nearest Neighborhood	KNN
Support Vector Machine	SVM
Newborn Cry Diagnostic Systems	NCDS
Expiratory Cries	EXP
Voiced Inspiratory Cries	INSV
United Nations Children’s Fund	UNICEF
Linear Prediction Coding	LPC
Probabilistic Neural Network	PNN
Hyperparameter Optimization	HPO
Bayesian Hyperparameter Optimization	BHPO
Machine Learning	ML
Respiratory Distress Syndrome	RDS
Discrete Fourier Transform	DFT
Discrete Cosine Transform	DCT
Spectral Entropy	SEN
Probability Mass Function	PMF
Fast Fourier Transform	FFT
Spectral Centroid	SC
Fuzzy Entropy Selection of the features	FE Selection
Fuzzy Entropy	FE
True Positive Rate	TPR
Predictive Positive Value	PPV
Matthews Correlation Coefficient	MCC

## References

[1] UNICEF, WHO, World Bank Group, United Nations Levels and Trends in Child Mortality. https://www.unicef.org/reports/levels-and-trends-child-mortality-report-2020.

[2] Fort A., Manfredi C. (1998). Acoustic analysis of newborn infant cry signals. Med. Eng. Phys..

[3] Michelsson K., SirviÖ P., Wasz-Höckert O. (1977). Pain cry in full-term asphyxiated newborn infants correlated with late findings. Acta Pædiatrica.

[4] Matikolaie F.S., Tadj C. (2020). On the use of long-term features in a newborn cry diagnostic system. Biomed. Signal Process. Control.

[5] Abou-Abbas L., Tadj C., Fersaie H.A. (2017). A fully automated approach for baby cry signal segmentation and boundary detection of expiratory and inspiratory episodes. J. Acoust. Soc. Am..

[6] Farsaie Alaie H., Tadj C. (2012). Cry-based classification of healthy and sick infants using adapted boosting mixture learning method for gaussian mixture models. Model. Simul. Eng..

[7] Messaoud A., Tadj C. Analysis of acoustic features of infant cry for classification purposes. Proceedings of the 2011 24th Canadian Conference on Electrical and Computer Engineering (CCECE).

[8] Kheddache Y., Tadj C. (2013). Acoustic measures of the cry characteristics of healthy newborns and newborns with pathologies. J. Biomed. Sci. Eng..

[9] Kheddache Y., Tadj C. (2013). Frequential characterization of healthy and pathologic newborns cries. Am. J. Biomed. Eng..

[10] Bano S., RaviKumar K. Decoding baby talk: A novel approach for normal infant cry signal classification. Proceedings of the 2015 International Conference on Soft-Computing and Networks Security (ICSNS).

[11] Parga J.J., Lewin S., Lewis J., Montoya-Williams D., Alwan A., Shaul B., Han C., Bookheimer S.Y., Eyer S., Dapretto M. (2020). Defining and distinguishing infant behavioral states using acoustic cry analysis: Is colic painful?. Pediatric Res..

[12] Abou-Abbas L., Alaie H.F., Tadj C. (2015). Automatic detection of the expiratory and inspiratory phases in newborn cry signals. Biomed. Signal Process. Control.

[13] Torres R., Battaglino D., Lepauloux L. Baby cry sound detection: A comparison of hand crafted features and deep learning approach. Engineering Applications of Neural Networks, Proceedings of the International Conference on Engineering Applications of Neural Networks, Athens, Greece, 25–27 August 2017.

[14] Satar M., Cengizler C., Hamitoglu S., Ozdemir M. (2022). Audio analysis based diagnosis of hypoxic ischemic encephalopathy in newborns. Int. J. Adv. Biomed. Eng..

[15] Orlandi S., Manfredi C., Bocchi L., Scattoni M.L. Automatic newborn cry analysis: A non-invasive tool to help autism early diagnosis. Proceedings of the 2012 Annual International Conference of the IEEE Engineering in Medicine and Biology Society.

[16] Zabidi A., Mansor W., Khuan L.Y., Yassin I.M., Sahak R. Classification of infant cries with hypothyroidism using multilayer perceptron neural network. Proceedings of the 2009 IEEE International Conference on Signal and Image Processing Applications.

[17] Kheddache Y., Tadj C. (2019). Identification of diseases in newborns using advanced acoustic features of cry signals. Biomed. Signal Process. Contro..

[18] Massengill R.M. (1969). Cry characteristics in cleft-palate neonates. J. Acoust. Soc. Am..

[19] Zabidi A., Mansor W., Lee K.Y. (2017). Optimal feature selection technique for mel frequency cepstral coefficient feature extraction in classifying infant cry with asphyxia. Indones. J. Electr. Eng. Comput. Sci..

[20] Wahid N., Saad P., Hariharan M. (2016). Automatic infant cry classification using radial basis function network. J. Adv. Res. Appl. Sci. Eng. Technol..

[21] Jam M.M., Sadjedi H. Identification of hearing disorder by multi-band entropy cepstrum extraction from infant’s cry. Proceedings of the 2009 International Conference on Biomedical and Pharmaceutical Engineering.

[22] Kheddache Y., Tadj C. (2015). Resonance frequencies behavior in pathologic cries of newborns. J. Voice.

[23] Liu L., Li W., Wu X., Zhou B.X. (2019). Infant cry language analysis and recognition: An experimental approach. IEEE/CAA J. Autom. Sin..

[24] Matikolaie F.S., Kheddache Y., Tadj C. (2022). Automated newborn cry diagnostic system using machine learning approach. Biomed. Signal Process. Control.

[25] Hariharan M., Saraswathy J., Sindhu R., Khairunizam W., Yaacob S. (2012). Infant cry classification to identify asphyxia using time-frequency analysis and radial basis neural networks. Expert Syst. Appl..

[26] Vaishnavi V., Dhanaselvam P.S. An automatic approach to extract features from the infant’s cry signals. Proceedings of the International Conference on Recent Trends in Computing, Communication & Networking Technologies (ICRTCCNT).

[27] Lahmiri S., Tadj C., Gargour C., Bekiros S. (2021). Characterization of infant healthy and pathological cry signals in cepstrum domain based on approximate entropy and correlation dimension. Chaos Solitons Fractals.

[28] Chang C.-Y., Chang C.-W., Kathiravan S., Lin C., Chen S.-T. (2017). DAG-SVM based infant cry classification system using sequential forward floating feature selection. Multidimens. Syst. Signal Process..

[29] Osmani A., Hamidi M., Chibani A. Machine learning approach for infant cry interpretation. Proceedings of the 2017 IEEE 29th International Conference on Tools with Artificial Intelligence (ICTAI).

[30] Oren A., Matzliach A., Cohen R., Friedman H. Cry-based detection of developmental disorders in infants. Proceedings of the 2016 IEEE International Conference on the Science of Electrical Engineering (ICSEE).

[31] Lakatos S. (2000). A common perceptual space for harmonic and percussive timbres. Percept. Psychophys..

[32] Kulkarni N., Bairagi V. (2017). Extracting salient features for EEG-based diagnosis of Alzheimer’s disease using support vector machine classifier. IETE J. Res..

[33] Alaie H.F., Abou-Abbas L., Tadj C. (2016). Cry-based infant pathology classification using GMMs. Speech Commun..

[34] Chang C.-Y., Hsiao Y.-C., Chen S.-T. Application of incremental SVM learning for infant cries recognition. Proceedings of the 2015 18th International Conference on Network-Based Information Systems.

[35] Rosales-Pérez A., Reyes-García C.A., Gonzalez J.A., Reyes-Galaviz O.F., Escalante H.J., Orlandi S. (2015). Classifying infant cry patterns by the genetic selection of a fuzzy model. Biomed. Signal Process. Control.

[36] Fuhr T., Reetz H., Wegener C. (2015). Comparison of supervised-learning models for infant cry classification/Vergleich von Klassifikationsmodellen zur Säuglingsschreianalyse. Int. J. Health Prof..

[37] Matikolaie F.S., Tadj C. (2022). Machine learning-based cry diagnostic system for identifying septic newborns. J. Voice.

[38] King R.D., Feng C., Sutherland A. (1995). Statlog: Comparison of classification algorithms on large real-world problems. Appl. Artif. Intell. Int. J..

[39] Michie D., Spiegelhalter D.J., Taylor C.C. (1994). Machine Learning, Neural and Statistical Classification.

[40] Kohavi R., John G.H. (1995). Automatic parameter selection by minimizing estimated error. Machine Learning Proceedings 1995.

[41] Mantovani R.G., Horváth T., Cerri R., Vanschoren J., de Carvalho A.C. Hyper-parameter tuning of a decision tree induction algorithm. Proceedings of the 2016 5th Brazilian Conference on Intelligent Systems (BRACIS).

[42] Olson R.S., Cava W.L., Mustahsan Z., Varik A., Moore J.H. Data-driven advice for applying machine learning to bioinformatics problems. Proceedings of the Pacific Symposium on Biocomputing 2018: Proceedings of the Pacific Symposium.

[43] Jaganathan P., Kuppuchamy R. (2013). A threshold fuzzy entropy based feature selection for medical database classification. Comput. Biol. Med..

[44] Luukka P. (2011). Feature selection using fuzzy entropy measures with similarity classifier. Expert Syst. Appl..

[45] Lee H.-M., Chen C.-M., Chen J.-M., Jou Y.-L. (2001). An efficient fuzzy classifier with feature selection based on fuzzy entropy. IEEE Trans. Syst. Man Cybern. Part B (Cybern.).

[46] Lohrmann C., Luukka P., Jablonska-Sabuka M., Kauranne T. (2018). A combination of fuzzy similarity measures and fuzzy entropy measures for supervised feature selection. Expert Syst. Appl..

[47] Khushaba R.N., Al-Jumaily A., Al-Ani A. Novel feature extraction method based on fuzzy entropy and wavelet packet transform for myoelectric control. Proceedings of the 2007 International Symposium on Communications and Information Technologies.

[48] Ruiz-Contreras J., Urquía L., Bastero R. (1999). Persistent crying as predominant manifestation of sepsis in infants and newborns. Pediatric Emerg. Care.

[49] Singh M., Gray C.P. (2018). Neonatal Sepsis.

[50] Moorman J.R., Delos J.B., Flower A.A., Cao H., Kovatchev B.P., Richman J.S., Lake D.E. (2011). Cardiovascular oscillations at the bedside: Early diagnosis of neonatal sepsis using heart rate characteristics monitoring. Physiol. Meas..

[51] Balayan S., Chauhan N., Chandra R., Kuchhal N.K., Jain U. (2020). Recent advances in developing biosensing based platforms for neonatal sepsis. Biosens. Bioelectron..

[52] Abou-Abbas L., Tadj C., Gargour C., Montazeri L. (2017). Expiratory and inspiratory cries detection using different signals’ decomposition techniques. J. Voice.

[53] Reby D., Levréro F., Gustafsson E., Mathevon N. (2016). Sex stereotypes influence adults’ perception of babies’ cries. BMC Psychol..

[54] Lester B.M., Boukydis C.Z. (1985). Infant Crying: Theoretical and Research Perspectives.

[55] Aucouturier J.-J., Nonaka Y., Katahira K., Okanoya K. (2011). Segmentation of expiratory and inspiratory sounds in baby cry audio recordings using hidden Markov models. J. Acoust. Soc. Am..

[56] Wasz-Hockert O., Lind J., Partanen T., Valanne E., Vuorenkoski V. (1968). The Infant Cry: A Spectrographic and Auditory Analysis.

[57] Huang X., Acero A., Hon H.-W., Foreword By-Reddy R. (2001). Spoken Language Processing: A Guide to Theory, Algorithm, and System Development.

[58] Brent W. (2010). Physical and Perceptual Aspects of Percussive Timbre. Ph.D. Thesis.

[59] Porta A., De Maria B., Bari V., Marchi A., Faes L. (2016). Are nonlinear model-free conditional entropy approaches for the assessment of cardiac control complexity superior to the linear model-based one?. IEEE Trans. Biomed. Eng..

[60] Magagnin V., Bassani T., Bari V., Turiel M., Maestri R., Pinna G.D., Porta A. (2011). Non-stationarities significantly distort short-term spectral, symbolic and entropy heart rate variability indices. Physiol. Meas..

[61] Young S., Evermann G., Gales M., Hain T., Kershaw D., Liu X., Moore G., Odell J., Ollason D., Povey D. (2002). The HTK Book.

[62] Cohen L. (1995). Time-Frequency Analysis.

[63] Benesty J., Sondhi M.M., Huang Y. (2007). Springer Handbook of Speech Processing.

[64] Misra H., Ikbal S., Bourlard H., Hermansky H. Spectral entropy based feature for robust ASR. Proceedings of the 2004 IEEE International Conference on Acoustics, Speech, and Signal Processing.

[65] Toh A.M., Togneri R., Nordholm S. (2005). Spectral entropy as speech features for speech recognition. Proc. PEECS.

[66] Kulkarni N., Bairagi V. (2018). EEG-Based Diagnosis of Alzheimer Disease: A Review and Novel Approaches for Feature Extraction and Classification Techniques.

[67] Almeida A., Schubert E., Smith J., Wolfe J. (2017). Brightness scaling of periodic tones. Atten. Percept. Psychophys..

[68] Weiss S.L., Pomerantz W.J., Torrey S.B., Kaplan S.L., Randolph A.G., Torrey S.B., Kaplan S.L. (2019). Septic shock in children: Rapid recognition and initial resuscitation (first hour). U: UpToDate.

[69] Amaro-Camargo E., Reyes-García C.A. (2007). Applying statistical vectors of acoustic characteristics for the automatic classification of infant cry. Advanced Intelligent Computing Theories and Applications-with Aspects of Theoretical and Methodological Issues, Proceedings of the International Conference on Intelligent Computing, Qingdao, China, 21–24 August 2007.

[70] Trivedi M.M., Bezdek J.C. (1986). Low-level segmentation of aerial images with fuzzy clustering. IEEE Trans. Syst. Man Cybern..

[71] Wu X., Kumar V., Quinlan J.R., Ghosh J., Yang Q., Motoda H., McLachlan G.J., Ng A., Liu B., Philip S.Y. (2008). Top 10 algorithms in data mining. Knowl. Inf. Syst..

[72] Latifpour H., Mosleh M., Kheyrandish M. (2015). An intelligent audio watermarking based on KNN learning algorithm. Int. J. Speech Technol..

[73] Sahak R., Mansor W., Lee K.Y., Zabidi A., Yassin A.I. (2013). Optimization of principal component analysis and support vector machine for the recognition of infant cry with asphyxia. Int. J. Comput. Appl..

[74] Feurer M., Hutter F. (2019). Hyperparameter optimization. Automated Machine Learning.

[75] Ashwini K., Vincent P.D.R. (2022). A deep convolutional neural network based approach for effective neonatal cry classification. Recent Adv. Comput. Sci. Commun. (Former. Recent Pat. Comput. Sci.).

[76] Parikh R., Mathai A., Parikh S., Sekhar G.C., Thomas R. (2008). Understanding and using sensitivity, specificity and predictive values. Indian J. Ophthalmol..

[77] Chicco D., Jurman G. (2020). The advantages of the Matthews correlation coefficient (MCC) over F1 score and accuracy in binary classification evaluation. BMC Genom..

[78] Vihinen M. (2012). How to evaluate performance of prediction methods? Measures and their interpretation in variation effect analysis. BMC Genom..

